# Chlorpheniramine Maleate Displaying Multiple Modes of Antiviral Action Against SARS-CoV-2: An Initial Mechanistic Study

**DOI:** 10.7759/cureus.92375

**Published:** 2025-09-15

**Authors:** Yaseen A Elshaier, Ahmed Mostafa, Joshua Costin, Syed A A Rizvi, Dennis J Pavón-Varela, Kristhel Gaitán-Zambrano, César Alas-Pineda

**Affiliations:** 1 Organic and Medical Chemistry Department, University of Sadat City, Sadat City, EGY; 2 Center of Scientific Excellence for Influenza Viruses, National Research Centre, Giza, EGY; 3 Medical Education Department, Nova Southeastern University Dr. Kiran C. Patel College of Allopathic Medicine, Fort Lauderdale, USA; 4 Biomedical Sciences Department, Larkin University, Miami, USA; 5 Research and Development Department, Dr. Ferrer Biopharma, Florida, USA

**Keywords:** adsorption interference, antiviral, cheminformatics, chlorpheniramine maleate, replication interference, sars-cov-2

## Abstract

Background

Chlorpheniramine maleate (CPM) has been identified as a potential antiviral compound against severe acute respiratory syndrome coronavirus 2 (SARS-CoV-2).

Materials and methods

The antiviral activity of CPM against SARS-CoV-2 was evaluated in vitro using Vero E6 cells. Cytotoxicity and antiviral assays assessed effects on viral adsorption, replication, and virucidal activity. Molecular docking was conducted to explore interactions with viral proteins and the angiotensin-converting enzyme 2 receptor.

Results

Our findings demonstrate that CPM exhibits antiviral properties by interfering with viral adsorption and replication, as well as directly inactivating the virus. Molecular docking analysis revealed interactions between CPM and essential viral proteins, such as the main protease receptor, spike protein receptor, and RNA polymerase. CPM's interactions were primarily hydrophobic in nature, with an additional hydrogen bond formation in the RNA polymerase active site.

Conclusions

These results suggest that CPM has the potential to serve as a multitarget antiviral agent against SARS-CoV-2 and potentially other respiratory viruses. Further investigations are warranted to explore its clinical implications and assess its efficacy in vivo.

## Introduction

Since the emergence of severe acute respiratory syndrome coronavirus 2 (SARS-CoV-2) in Wuhan, China, a few drugs have been approved for the treatment and prophylaxis of coronavirus disease 2019 (COVID-19) [1]. Various attempts have been made to identify and repurpose currently approved medications for the treatment of COVID-19, specifically those with antiviral potential, such as the antihistamine drugs hydroxyzine, azelastine, carbinoxamine maleate, and chlorpheniramine maleate (CPM) [1-3]. CPM is a first-generation H1 antihistamine that has been a longstanding treatment option for allergies, hay fever, the common cold, cough, and nasal congestion. Its anti-inflammatory and immunomodulatory properties likely contribute to its efficacy [4,5]. It is worth noting that several studies have demonstrated CPM's antiviral activity against respiratory viruses, including influenza and SARS-CoV-2 [3,6-8]. Furthermore, early clinical studies have demonstrated CPM's effectiveness in treating COVID-19, not only improving recovery and reducing hospitalisations but also showing safety and efficacy when administered intranasally, both for COVID-19 and allergic rhinitis, which supports its potential as an early and practical intervention therapy [6-11]. Our research team has also reported on the safety and efficacy of intranasally administered CPM for the treatment of COVID-19 and allergic rhinitis [9-12].

Mechanistically, hydroxyzine and related antihistamines can inhibit SARS-CoV-2 entry via off-target inhibitory angiotensin-converting enzyme 2 (ACE2) activity by forming intermolecular interactions with the active site, as well as virus replication by binding to the sigma-1 receptor [2]. While the mode of action of CPM against influenza is well established, the mechanisms underlying its antiviral effect against SARS-CoV-2, including the corresponding cellular compartments and molecular targets, remain unknown. We hypothesized that CPM exerts multi-modal antiviral activity against SARS-CoV-2. To test this hypothesis, our primary aim was to assess in vitro antiviral efficacy across adsorption, replication, and virucidal stages, and our secondary aim was to explore molecular interactions via docking.

This article was previously posted to the bioRxiv preprint server on August 29, 2023 (doi: https://doi.org/10.1101/2023.08.28.554806).

## Materials and methods

Cells and virus strain

Vero E6 cells were cultured in Dulbecco's modified Eagle's medium (DMEM) (Invitrogen, Germany) supplemented with 10% fetal bovine serum (FBS) and 1% penicillin/streptomycin at 37°C in 5% CO₂. The hCoV-19/Egypt/NRC-3/2020 "NRC-03-nhCoV" strain (clade A2a) was propagated and titrated as previously described [7,9,13]. All experiments with live SARS-CoV-2 were conducted under biosafety level-3 conditions.

MTT cytotoxicity assay

To determine the half-maximal cytotoxic concentration (CC_50_), stock solutions of CPM were prepared in 10% dimethyl sulfoxide with double-distilled water and diluted in DMEM. Cytotoxicity was evaluated by the MTT assay, with minor modifications from established protocols [14-16]. Vero E6 cells were seeded in 96-well plates (3 × 10⁵ cells/mL, 100 µL/well) and incubated for 24 hours at 37°C, 5% CO₂. Cells were then treated in triplicate with varying CPM concentrations for 24 hours, washed with PBS, and incubated with MTT solution (20 µL of 5 mg/mL) for four hours. Formazan crystals were dissolved in 200 µL of acidified isopropanol. Absorbance was measured at 540 nm (reference 620 nm) using an Anthos Zenyth 200RT plate reader (Biochrom Ltd., Cambridge, UK). Untreated cells and blanks were included as controls. CC_50_ values were calculated by nonlinear regression of log concentration versus normalized response using GraphPad Prism version 5.01 (Dotmatics, Boston, MA, USA).

Mode of anti-SARS-CoV-2 action

The potential effects of CPM on viral adsorption, replication, and direct virucidal activity were investigated. These assays were designed to directly address our hypothesis that CPM exhibits activity at multiple stages of the viral life cycle.

Adsorption mechanism

Viral adsorption was evaluated by plaque reduction assay with minor modifications [14]. Vero E6 cells were seeded in six-well plates to 80-90% confluence. Uninfected control cells were included in each plate. Cells were incubated with CPM (156, 78, 39, or 19 µg/mL) at 4°C for one hour, then exposed to SARS-CoV-2 (7.5 × 10⁵ PFU/mL) for one hour at 37°C. An overlay of DMEM with 2% agarose, 0.2% bovine serum albumin, and 1% antibiotic-antimycotic was applied. After three days, plaques were fixed with 10% formalin and stained with crystal violet. Viral inhibition was calculated relative to untreated controls. No benchmark antiviral such as remdesivir was available during this study; instead, untreated infected and uninfected cells were used as infection and negative controls, and published remdesivir/nirmatrelvir data are referenced for context.

Replication mechanisms

To assess replication, Vero E6 cells were infected with SARS-CoV-2 (7.5 × 10⁵ PFU/mL) for one hour, washed with PBS, and treated with CPM (156, 78, 39, or 19 µg/mL) for one hour. Cells were then overlaid with DMEM containing 2% agarose and 1% antibiotic-antimycotic and incubated for three days. Plaques were fixed and stained, and inhibition percentages were determined relative to controls [17].

Virucidal mechanism

The virucidal effect was tested by incubating SARS-CoV-2 (7.5 × 10⁵ PFU/mL) with CPM at inhibitory concentrations for one hour. The mixture was serially diluted and applied to Vero E6 cells. Following one hour of adsorption, cells were overlaid with DMEM and incubated for plaque development. Plaque reduction relative to the untreated virus was used to calculate virucidal activity [15].

Chemoinformatics and molecular modelling

X-ray crystal structures of SARS-CoV-2 main protease (Mpro, PDB IDs 6yef, 6lu7), spike (S) glycoprotein (PDB ID 6vsb), RNA polymerase (PDB ID 6m71), and ACE2 receptor (PDB ID 1r42) were retrieved from the Protein Data Bank. Docking was performed using OpenEye Scientific Software version 2.2.5 (Santa Fe, NM, USA). Validation was confirmed by re-docking co-crystallized ligands, with RMSD values confirming docking accuracy [18]. Binding energies are reported as qualitative indicators only; results are hypothesis-generating and not confirmatory.

Physicochemical parameters and lipophilicity calculations

Drug parameters, including ClogP, were calculated based on their reported values from the ChEMBL, DrugBank, and PubChem websites, all of which are free-access resources. Lipinski's rule (also known as the Rule of Five) was calculated using the free access to the website https://www.molsoft.com/servers.html.

Statistical analysis

All experimental results are expressed as mean ± standard deviation (SD). The 50% CC_50_ and the percentage of viral inhibition were determined using nonlinear regression analysis with GraphPad Prism version 5.01. Differences between groups were analyzed using one-way analysis of variance followed by Tukey’s post hoc test. A p-value of less than 0.05 was considered statistically significant.

## Results

**Figure 1:**
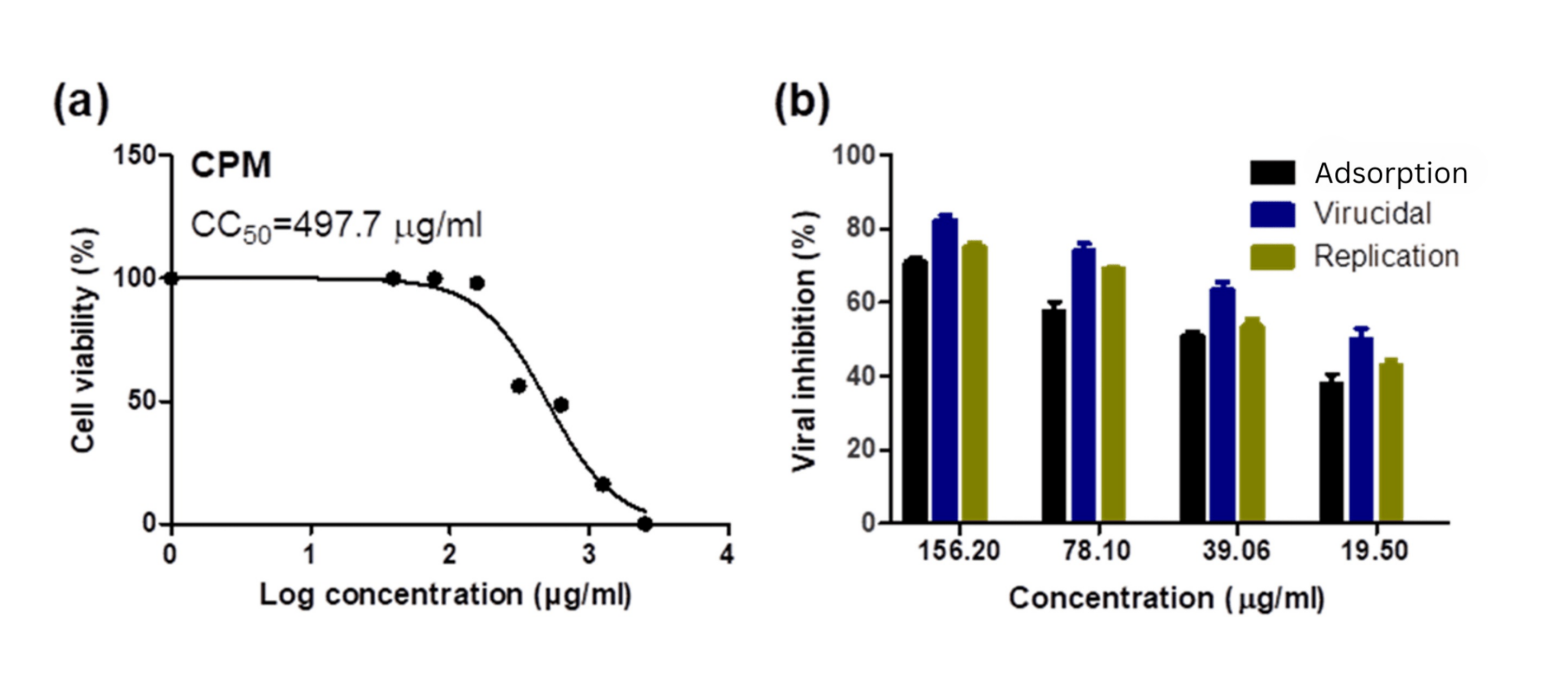
Cytotoxicity and cellular compartment mode of antiviral action of CPM in Vero E6 cells against SARS-CoV-2 (A) Cytotoxicity of the tested CPM in Vero E6 cells. The cytotoxicity of CPM based on the dose-response was determined using MTT. The CC_50_ was calculated for each compound using nonlinear regression analysis in GraphPad Prism version 5.01. (B) Mode of anti-SARS-CoV-2 action of CPM. Virucidal, viral replication inhibition, and viral adsorption inhibition mechanisms were studied for CPM at different concentrations using the plaque reduction assay. CPM: chlorpheniramine maleate, MTT: 3-(4,5-dimethylthiazol-2-yl)-2,5-diphenyltetrazolium bromide, CC_50_: cytotoxic concentration

The half-maximal cytotoxic concentration (CC_50_) of the tested CPM in Vero E6 cells was 497.7 µg/ml (Figure 1a). The mode of anti-SARS-CoV-2 action indicates that CPM exerts dose-dependent antiviral effects via multiple mechanisms, including a direct virucidal impact, viral replication inhibition, and viral adsorption inhibition (Figure 1b).

**Figure 2:**
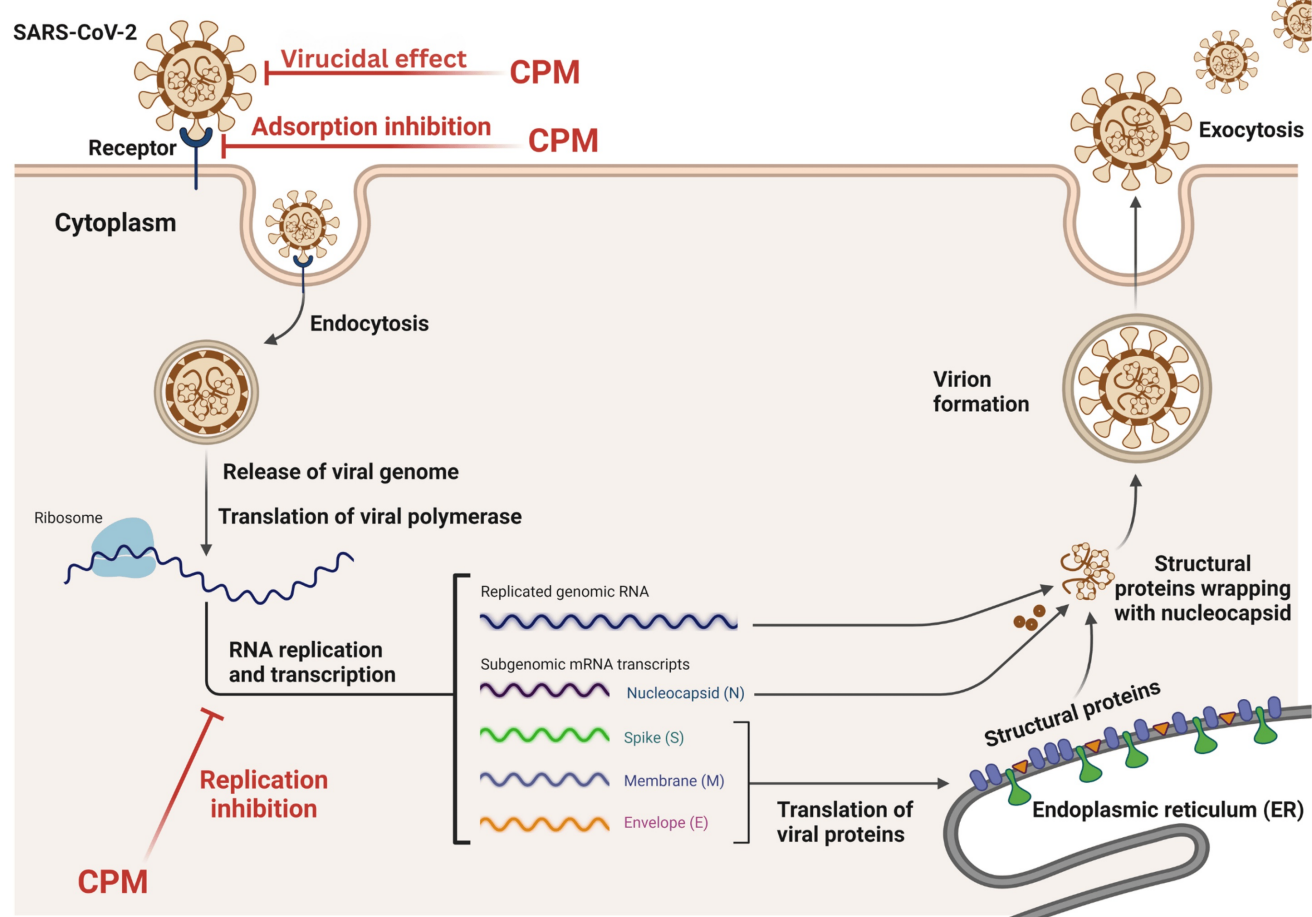
Model for SARS-CoV-2 antiviral mechanisms mediated by CPM adapted and modified from SARS-CoV-2: severe acute respiratory syndrome coronavirus 2, CPM: chlorpheniramine maleate, RNA: ribonucleic acid This figure was created using BioRender.com.

The suggested model for the antiviral mode of CPM is shown in Figure 2.

**Figure 3:**
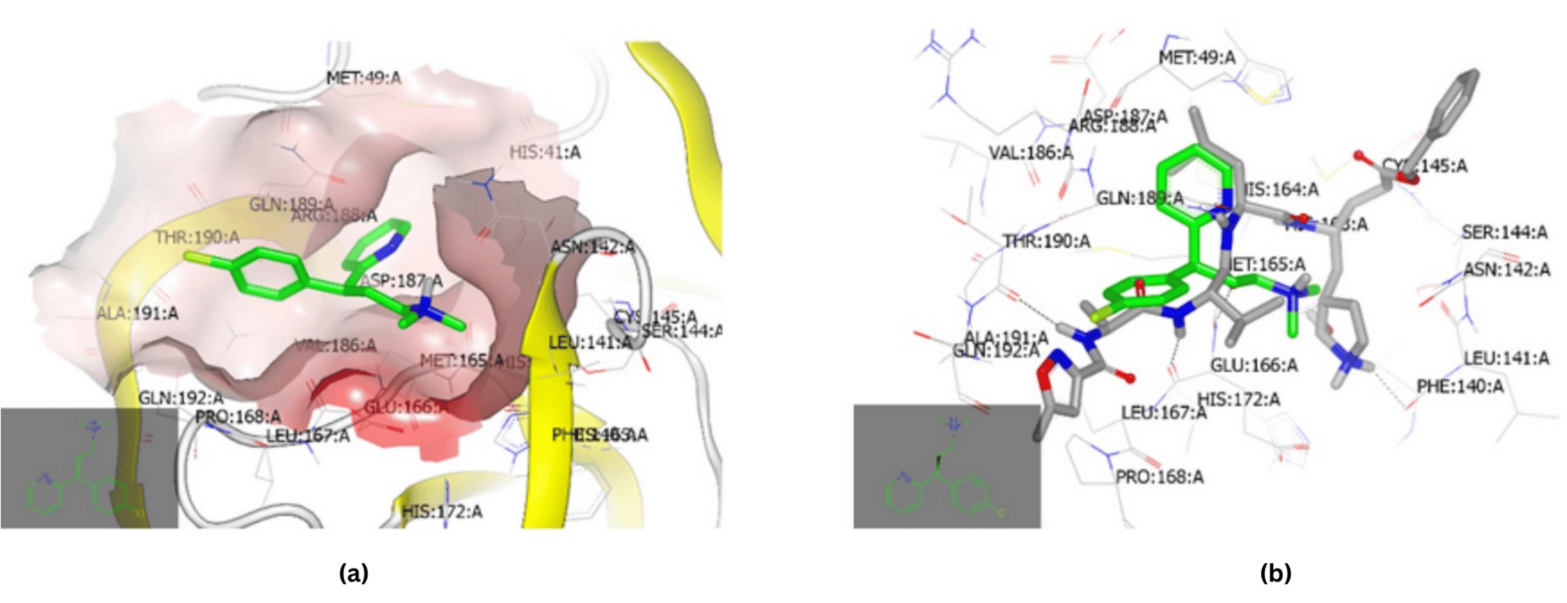
Molecular docking of CPM with SARS-CoV-2 Mpro (A) CPM formed an interaction with the receptor of Mpro (PDB ID:6lu7) of SARS-CoV-2 through a hydrophobic-hydrophobic interaction. (B) CPM (green color) overlaid with N3, the co-crystallized ligand of Mpro (PDB ID:6lu7) from SARS-CoV-2 (grey color). CPM: chlorpheniramine maleate, SARS-CoV-2: severe acute respiratory syndrome coronavirus 2, Mpro: main protease

The analysis of the CPM's binding mode revealed interactions with the receptor of Mpro (PDB ID:6lu7) through a hydrophobic-hydrophobic interaction (Figure 3a). From Figure 3b, the CPM (green color) is overlaid with N3, the co-crystallized ligand (grey color).

**Figure 4:**
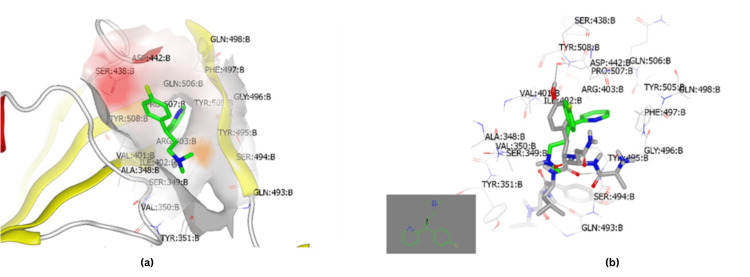
Molecular docking of CPM with SARS-CoV-2 S protein (A) CPM formed an interaction with the receptor of the S protein (PDB ID:6vsb) of SARS-CoV-2 through a hydrophobic-hydrophobic interaction. (B) CPM (green color) showed overlay with the co-crystallized ligand S protein (PDB ID:6vsb) of SARS-CoV-2 (grey color) with little similarity in binding pose and mode. CPM: chlorpheniramine maleate, SARS-CoV-2: severe acute respiratory syndrome coronavirus 2, S: spike

Docking of CPM with S glycoprotein (PDBID:6vsb) presented a hydrophobic-hydrophobic interaction (Figure 4a). The analysis of the binding mode and pose of this drug (green color) in comparison to the co-crystallized ligand (grey) is shown in Figure 4b. The P-chlorophenyl ring is overlaid with the P-methoxy moiety and the N, N-dimethylethanolamine part.

**Figure 5:**
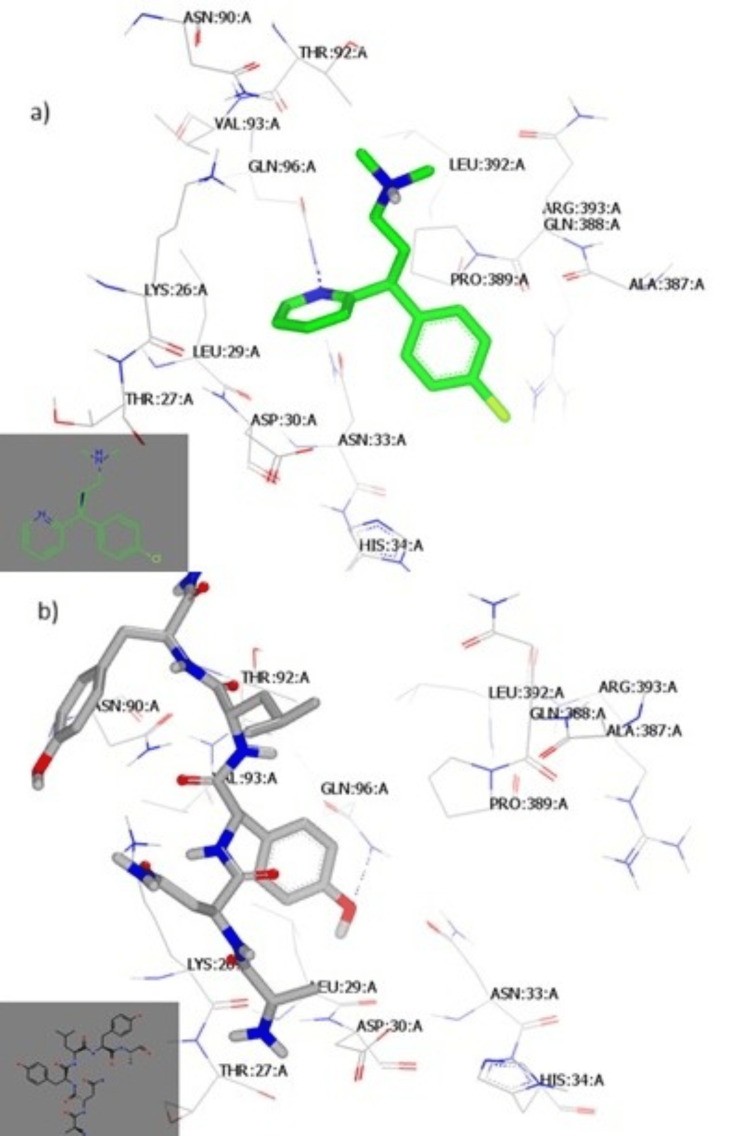
(a) Molecular docking of CPM with ACE2 receptor (ID 1R42). (b) Molecular docking of standard ligand for SARS-CoV-2 S protein with ACE2 receptor (ID 1R42) CPM: chlorpheniramine maleate, SARS-CoV-2: severe acute respiratory syndrome coronavirus 2, ACE2: angiotensin-converting enzyme 2, S: spike

ACE2 is a functional receptor that allows SARS-CoV-2 to enter human lung cells via its S protein. Molecular docking with ACE receptors was then performed. Docking of CPM with the ACE2 receptor (PDB:ID1R42) showed that the protonated tertiary amine of chlorpheniramine at physiological pH participated in hydrogen bond (HB) formation with Gln 96, as shown in Figure 5a [16]. This ACE2 docking is presented as exploratory; our primary focus remains on viral targets (Mpro, S, and RdRp). However, CPM does not interact with the same domain as the co-crystallized ligand. The standard S protein-ligand was also docked and illustrated HB formation with the same amino acid (Figure 5b) [17].

**Figure 6:**
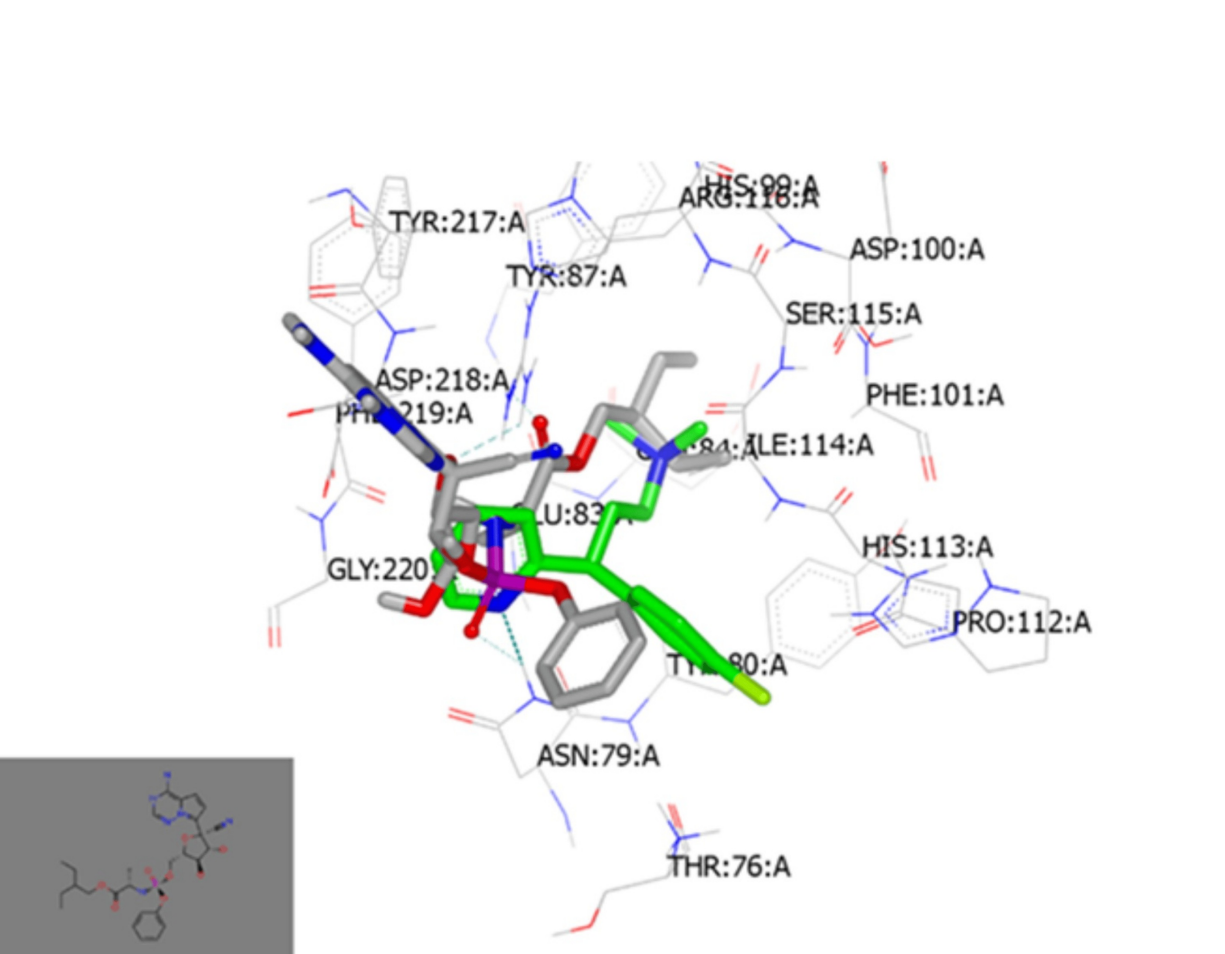
Molecular docking of CPM with SARS-CoV-2 RNA polymerase Visual representation using the Vida application: (a) CPM interacted with ID:6m71 active site by forming HB with Asn:79 A through the nitrogen atom of the pyridine moiety. CPM: chlorpheniramine maleate, SARS-CoV-2: severe acute respiratory syndrome coronavirus 2, RNA: ribonucleic acid, HB: hydrogen bond

Docking with RNA polymerase (PDB ID:6m71) showed that CPM formed an HB with Asn:79 A through the nitrogen atom of the pyridine moiety. The ethylamine derivative is deeply embedded inside the pocket of the active site, as shown in Figure 6.

According to previous molecular docking, in vitro viral inactivation, and molecular mechanisms, CPM can inhibit virus replication. Regarding this, I will implement lipophilic metric studies in comparison to the reported drug remdesivir (RNA polymerase inhibitor, antiviral replication). Lipophilic metric analysis, particularly ligand efficiency (LE) and ligand lipophilic efficiency (LLE) scores, is crucial in the drugability process and during the drug repositioning strategy.

The ligand-target interaction is the primary parameter in the drug discovery and development pipeline. CMP inhibits SARS-CoV-2 through multiple mechanisms, including viral absorption, replication, and virucidal effects.

The challenge in drug discovery is to improve activity while maintaining lipophilicity, thereby avoiding "molecular obesity" during drug development. An acceptable lead drug should have an LLE value ≥ 3, while an LLE value ≥ 5 is recommended for a drug-like candidate.

As illustrated in Table 1, CPM displayed LE better than remdesivir (0.39 vs. 0.17, respectively), while remdesivir showed LEE with a value slightly higher than CPM (2.64 vs. 2.26, respectively). CPM can penetrate the blood-brain barrier (BBB) more than remdesivir, as shown in Table 1. CPM has a Lipinski rule of five lower than remdesivir.

**Table 1:** Selected physicochemical properties and LE scores of CP and remdesivir against SARS-CoV-2 NHA: non-hydrogen atom (heavy atom), HBA: hydrogen bond acceptor, HBD: hydrogen bond donor, RO5: rule of thumb to evaluate drug-likeness or determine if a chemical compound with a particular pharmacological or biological activity has chemical and physical properties that would make it likely to be orally active, tPSA: topological polar surface area, LE: ligand efficiency, LLE: ligand lipophilic efficiency, BBB: blood-brain barrier

	Rule of five (RO5)				No. of rotatable bond	tPSA (A^0^)	BBB	CLogP	Experimental data			
	Molecular wt	NHA	HBA	HBD					IC_50_ (µM)	pIC_50_	LE	LLE
CPM	274.12	19	2	0	5	12.88	5.5	3.18	3.6	5.44	0.39	2.26
Remdesivir	602.23	42	12	5	14	160.14	1.02	2.44	8.24	5.08	0.17	2.64

## Discussion

Herein, we investigated the in vitro impact of CPM treatment on the three main compartments during the SARS-CoV-2 replication cycle, including its direct-acting virucidal effect, antagonism of viral entry by interfering with viral adsorption, and inhibition of viral replication within the host cell. These findings support, but do not prove, our initial hypothesis; they provide preliminary evidence that requires confirmation in additional cell lines and in vivo models. These results suggest CPM may act as a versatile antiviral candidate with clinical potential. These docking findings should be interpreted qualitatively, as we did not perform quantitative free energy simulations.

Mounting evidence indicates that CPM may have both antiviral and anti-inflammatory properties, which could be beneficial in treating COVID-19. Furthermore, molecular modelling data analyzed the antiviral activity of CPM, comparing the chemical structure of CPM with that of different over-the-counter drugs. CPM was structurally similar to medications known to have an anti-inflammatory effect and to medications known to have an antiviral effect in terms of drug-receptor interactions [6,16]. Clinical studies have demonstrated that CPM enhances clinical recovery and reduces hospitalizations in patients with COVID-19 [6,9-10]. In a two-part clinical study conducted by our team, ACCROS-I and ACCROS-II, a combined total of 110 patients with early COVID-19 received intranasal CPM [18-21]. The results showed a decrease in hospitalizations and fewer severe cases. The study provides evidence of the main mechanisms responsible for the antiviral actions of CPM against SARS-CoV-2. Based on the results, CPM shows potential to decrease viral load in patients; however, this requires confirmation in animal models and clinical studies.

Currently, two major antivirals have received emergency use authorization for COVID-19: Paxlovid, an oral protease inhibitor, and remdesivir, an intravenous RNA polymerase inhibitor. While these agents are effective in specific clinical contexts, CPM differs by being widely available, orally and intranasally deliverable, and potentially multitarget in its antiviral action, offering a distinct profile compared with single-mechanism drugs.

Furthermore, CPM has been shown to exhibit broad-spectrum antiviral effects, including against various strains of SARS-CoV-2 [3,8,10]. However, the present study focused on viral targets and not host-directed targets. The authors cannot rule out the possibility that other mechanisms, including host-targeted effects, may be involved in CPM’s antiviral effects, which require further transcriptomic and proteomic studies. These findings suggest CPM has the potential to decrease viral load in patients, but confirmation in animal models and clinical studies is required before any therapeutic conclusions can be drawn.

A strength of this study is its multi-step evaluation across adsorption, replication, and virucidal assays, complemented by chemoinformatics/docking. This combination enhances mechanistic interpretation and contextualizes our findings in relation to prior clinical evidence.

Limitations of the present study include the in vitro nature of the experiments, which may not fully replicate in vivo conditions. The absence of a positive control for all tested mechanisms restricts direct comparison with established antivirals. No benchmark antiviral such as remdesivir was available during this study; instead, untreated infected and uninfected cells were used as infection and negative controls, and published remdesivir/nirmatrelvir data are referenced for context. Furthermore, host-targeted effects were not explored, and additional transcriptomic and proteomic analyses are warranted to further investigate these findings. Due to biosafety and resource constraints, full dose-response curves for EC_50 _and selectivity index were not feasible; instead, we reported CC_50_ and concentration-dependent plaque reduction as preliminary indicators.

## Conclusions

CPM inhibits SARS-CoV-2 through multiple mechanisms, such as interference with viral adsorption, replication, and virucidal activity. These findings, considered alongside preliminary clinical observations suggesting symptom reduction and slower disease progression, support further evaluation of CPM as a potential multitarget antiviral. Beyond COVID-19, the antiviral properties of CPM suggest it could also be helpful against other respiratory viral pathogens. Its multitarget mechanism may also help reduce the emergence of viral resistance.

Given these advantages, randomized controlled trials are urgently needed to confirm the practical relevance of these findings. These trials should focus on determining optimal dosing regimens, evaluating combination therapies, and assessing clinical outcomes across diverse patient populations. If proven effective in these settings, CPM could be a cost-effective and widely accessible intervention to reduce the disease burden, hospitalizations, and healthcare resource utilization associated with COVID-19 and other viral respiratory infections.
